# Retinal Artery Occlusion and Incident Dementia in the All of Us Research Program: Detection Bias Calibration with Prespecified Negative-Control Outcomes

**DOI:** 10.14740/jocmr6628

**Published:** 2026-07-31

**Authors:** Meet Popatbhai Kachhadia, Piyush Puri, Usmaan Topiwala, Juber D. Shaikh, Gurnoor Gill, Shailesh Gupta, Michelle Byrus, Harshal A. Sanghvi

**Affiliations:** a Department of Neurology, Charles E. Schmidt College of Medicine, Florida Atlantic University, Boca Raton, FL, USA; b Department of Internal Medicine, Icahn School of Medicine at Mount Sinai, Queens, New York, NY, USA; c SMT NHL Municipal Medical College, Ahmedabad, Gujarat, India; d Department of Neurology, Henry Ford Hospital, Detroit, MI, USA; e Charles E. Schmidt College of Medicine, Florida Atlantic University, Boca Raton, FL, USA; f Specialty Retina Center, Deerfield Beach, FL, USA; g Department of Technology and Clinical Trials, Advanced Research LLC, Deerfield Beach, FL, USA; h Department of Information Technology and Operations Management (ITOM), College of Business, Florida Atlantic University, Boca Raton, FL, USA

**Keywords:** Retinal artery occlusion, Cerebrovascular disease, Dementia, Negative-control outcomes, Detection bias

## Abstract

**Background:**

Retinal artery occlusion (RAO) shares vascular pathophysiology with cerebrovascular disease, and its relationship to incident dementia remains unsettled. Reported associations may reflect shared vascular pathology, differential ascertainment along the intensive workup pathways that RAO patients enter, or both. No prior study has calibrated RAO–dementia estimates against prespecified negative-control outcomes spanning distinct ascertainment pathways.

**Methods:**

Among 129,279 All of Us participants aged 50 years or older and free of prevalent dementia (controlled-tier release R2024Q3R9, OMOP common data model), strict RAO was defined by seven verified SNOMED concept identifiers (n = 339). Incident dementia required two or more codes at least 30 days apart (Wilkinson algorithm), with prespecified vascular and Alzheimer subtypes. Cox models used age as timescale with left truncation and time-varying exposure; 1:5 propensity-score matching was the primary confounder-adjusted analysis. Cataract, benign paroxysmal positional vertigo (BPPV), and inguinal hernia were prespecified negative controls for ophthalmology, neurology, and general-contact pathways.

**Results:**

There were 1,506 incident dementia events. RAO was not associated with all-cause dementia (unmatched hazard ratio (HR) 0.81, 95% confidence interval (CI) 0.40–1.64; matched HR 1.33, 95% CI 0.72–2.47; 10 exposed events). The vascular dementia estimate remained elevated but imprecise (unmatched HR 1.93, 95% CI 1.02–3.66; matched HR 1.81, 95% CI 0.66–4.94; 3 exposed events). Cataract was elevated (HR 1.87, 95% CI 1.45–2.42), whereas BPPV (HR 1.27, 95% CI 0.90–1.79) and hernia (HR 1.10, 95% CI 0.68–1.78) were not.

**Conclusions:**

We found no evidence that RAO is independently associated with incident dementia, replicating a large European null finding in a diverse United States cohort. The cohort was underpowered to exclude clinically meaningful effects, particularly for vascular dementia. Detection bias in this dataset was demonstrably pathway-specific, but same-pathway calibration accounts for only part of the residual vascular dementia estimate.

## Introduction

Retinal artery occlusion (RAO) is an ischemic event of the central or branch retinal artery that shares its pathophysiologic substrate with cerebral small-vessel disease. The American Heart Association classifies central RAO as a stroke equivalent for evaluation and secondary prevention [1]. Given this shared vascular biology, RAO has been proposed as an early marker of vascular cognitive impairment.

The evidence is less consistent than is often assumed. The largest study of RAO as an isolated exposure, a Danish national cohort of 2,205,159 individuals aged 65 years or older including 8,863 with RAO, found no independent association with all-cause dementia after adjustment for systemic comorbidity (hazard ratio (HR) 0.98, 95% confidence interval (CI) 0.91–1.06), and a vascular dementia association that fell from 1.61 in the crude model to 1.12 (95% CI 0.95–1.33) once comorbidity was accounted for [2]. Studies reporting positive associations have generally examined retinal vein occlusion or composite retinal artery/vein occlusion exposures [3, 4]. A cross-sectional analysis of 37,208 individuals likewise concluded that the apparent association was secondary to shared risk factors, principally age and stroke [4].

Two methodological problems recur. First, vascular comorbidity adjustment has typically relied on narrow concept sets that under-capture true prevalence, leaving residual confounding. Second, and to our knowledge unaddressed, no RAO–dementia study has calibrated its estimates against negative-control outcomes to quantify differential ascertainment [5, 6]. Patients with RAO enter intensive vascular and ophthalmologic workup pathways. Each pathway increases the probability that pathway-specific incidental conditions are detected and coded, producing HRs that can resemble biological effects.

We tested whether RAO is independently associated with incident dementia in a diverse contemporary United States cohort, and used three prespecified negative-control outcomes spanning ophthalmology, neurology, and general healthcare contact to estimate the magnitude of pathway-specific detection bias operating in the same dataset. We hypothesized that any apparent association would attenuate after confounder adjustment, and that a same-pathway negative control would indicate how much of any residual estimate could be attributed to differential ascertainment.

## Materials and Methods

### Data source and cohort

The All of Us Research Program is a National Institutes of Health–funded prospective cohort with linked electronic health records (EHRs) harmonized to the Observational Medical Outcomes Partnership (OMOP) common data model [7, 8]. We used controlled-tier release R2024Q3R9. Cohort entry was the date of the first recorded clinical visit. Participants were eligible if they were aged 50 years or older at cohort entry, had at least one recorded visit, and had no dementia code recorded on or before cohort entry. Of 626,396 participants, 129,279 met all criteria. Follow-up ran from cohort entry to the first qualifying dementia event or the last recorded visit, whichever came first. Concept-set tables, full SQL, the STROBE checklist, and the Researcher Workbench notebook reference appear in the online supplement. All analyses reported here derive from a single final cohort build.

### Exposure

Strict RAO comprised seven SNOMED concept identifiers covering central, branch, partial, and unspecified retinal artery occlusion, each verified against the All of Us concept table. The broad definition added amaurosis fugax concepts as a prespecified sensitivity analysis. RAO events recorded before age 50 years were not counted as exposures. Central RAO (CRAO) and non-central RAO were prespecified subtypes for the cataract negative-control analysis.

### Outcomes

Incident dementia was defined using the Wilkinson algorithm, a validated case definition requiring two or more specific dementia concept codes recorded at least 30 days apart [9]. Requiring more than one code reduces misclassification from isolated codes entered during a single encounter for evaluation of possible cognitive complaints. We applied 11 verified SNOMED dementia concepts; prespecified subtypes were vascular dementia (three concepts) and Alzheimer disease (three concepts). SNOMED CT is the standardized clinical terminology to which All of Us EHR data are mapped within OMOP, and a concept identifier is a stable numeric label for a specific condition. The algorithm is deliberately specificity-favoring: it identified 1,506 incident cases from a substantially larger pool of any-dementia codes.

### Covariates

Hypertension, diabetes, hyperlipidemia, atrial fibrillation, heart failure, stroke, transient ischemic attack, depression, and chronic kidney disease were defined by ancestor expansion via the OMOP concept_ancestor table and were coded as present if the qualifying condition date fell on or before cohort entry. Sex was operationalized using All of Us gender-identity concepts (45878463 female, 45880669 male) as a proxy for sex, and the term sex is used consistently hereafter. Calendar year of cohort entry was included to address secular trends in coding intensity. Race, ethnicity, educational attainment, body mass index, smoking, alcohol use, and medication exposures were not included in the analytic dataset and were not modelled; this is addressed in the “Limitations”.

### Negative-control outcomes

Three negative-control outcomes were prespecified to span distinct ascertainment pathways: cataract (ophthalmology), BPPV (neurology), and inguinal hernia (general healthcare contact) [5, 6]. All used ancestor expansion, and participants with a prevalent code for the outcome in question at cohort entry were excluded (for example, 166 for BPPV; the cataract analysis included 127,356 participants). Each negative control was estimated with the identical model specification used for the unmatched adjusted primary analyses: Cox proportional hazards on an age timescale with left truncation at cohort entry, and the same covariate set listed above. Negative controls were not propensity-matched, and are therefore compared with the unmatched primary estimates.

### Statistical analysis

Cox proportional hazards models used age as the timescale with left truncation at cohort entry and time-varying RAO exposure. Propensity-score matching (1:5 nearest neighbor on the logit propensity score, caliper 0.2 SD) was the primary confounder-adjusted analysis; balance was assessed by standardized mean differences (SMDs) before and after matching. Prespecified sensitivity analyses were the broad RAO definition and, for vascular dementia, exclusion of participants with prevalent stroke. The proportional hazards assumption was assessed by a treatment-by-log(time) interaction, fitted in a 50:1 random sample of unexposed participants for computational tractability; Schoenfeld residual tests are not defined for left-truncated data in the software used. The cataract subtype analysis likewise used a 10:1 random sample of unexposed participants. E-values were computed per VanderWeele and Ding as a measure of robustness to unmeasured confounding [10]. Bonferroni correction was applied across five prespecified primary tests (α = 0.01), with Benjamini–Hochberg false discovery rate reported alongside. Analyses used Python lifelines 0.30 within the All of Us Researcher Workbench.

This retrospective cohort study analyzed de-identified EHR and survey data obtained through the All of Us Research Program Researcher Workbench. The study met criteria for exemption from Institutional Review Board oversight under the Common Rule (45 CFR 46.104[d]), and formal IRB review and approval were therefore not required. This study was conducted in accordance with applicable institutional and national ethical standards for research involving human participant data and adhered to the principles outlined in the Declaration of Helsinki.

## Results

### Cohort

Of 626,396 All of Us participants, 129,279 met inclusion criteria. The strict definition yielded 339 participants with RAO (CRAO 162; non-central RAO 177); the broad definition added 439 amaurosis fugax cases (n = 778). Mean age at cohort entry was 61.0 years (SD 6.8) in the RAO+ group and 60.4 years (SD 7.1) in the RAO− group (SMD 0.082). The two largest baseline imbalances were calendar year of cohort entry (mean 2008.2 versus 2012.7; SMD −0.726) and sex (35.7% versus 54.0% female; SMD −0.374); participants with RAO entered the cohort approximately 4.5 years earlier and were followed substantially longer (median 13.3 years, interquartile range (IQR) 9.0–19.0, versus 8.2 years, IQR 4.2–12.7). Baseline comorbidity was modestly higher in the RAO+ group, with all SMDs below 0.10 (Table 1). There were 1,506 incident Wilkinson-confirmed dementia events (RAO+ 10; RAO− 1,496), 220 vascular dementia events (RAO+ 3), and 447 Alzheimer disease events (RAO+ 4).

**Table 1:** Baseline Characteristics and Covariate Balance Before and After 1:5 Propensity-Score Matching, by Retinal Artery Occlusion Status

Characteristic	RAO+ (n = 339)	RAO− (n = 128,940)	SMD, pre-match	SMD, post-match
Age at cohort entry, mean (SD), years	61.0 (6.8)	60.4 (7.1)	0.082	−0.034
Calendar year of cohort entry, mean	2008.2	2012.7	−0.726	−0.015
Female, n (%)	121 (35.7)	69,595 (54.0)	−0.374	0.005
Hypertension, n (%)	65 (19.2)	19,944 (15.5)	0.098	−0.027
Diabetes, n (%)	31 (9.1)	8,372 (6.5)	0.099	0.010
Hyperlipidemia, n (%)	44 (13.0)	14,704 (11.4)	0.048	−0.005
Atrial fibrillation, n (%)	8 (2.4)	1,886 (1.5)	0.066	0.012
Heart failure, n (%)	8 (2.4)	1,707 (1.3)	0.077	0.051
Prior ischemic stroke, n (%)	3 (0.9)	656 (0.5)	0.045	0.000
Prior transient ischemic attack, n (%)	0 (0.0)	307 (0.2)	−0.069	0.000
Depression, n (%)	13 (3.8)	5,013 (3.9)	−0.003	−0.030
Chronic kidney disease, n (%)	7 (2.1)	1,703 (1.3)	0.058	−0.004
Follow-up, median (IQR), years	13.3 (9.0–19.0)	8.2 (4.2–12.7)	-	-

All covariates are defined at cohort entry. Post-match column refers to the 1:5 propensity-score matched set (339 exposed, 1,695 unexposed). All 339 exposed participants were matched; none were dropped under the caliper. IQR: interquartile range; RAO: retinal artery occlusion; SD: standard deviation; SMD: standardized mean difference.

### Matching performance

All 339 exposed participants were matched; none were dropped under the 0.2 SD caliper of the logit propensity score (caliper width 0.169), yielding 339 exposed and 1,695 unexposed participants (n = 2,034). The matched exposed group is therefore identical to the full exposed group. The propensity-score distribution of the exposed group (range 0.0003–0.0255) lay entirely within that of the unexposed group (range 0.0001–0.0437), indicating common support across the full exposed distribution. After matching, the largest absolute SMD across all covariates was 0.051 (heart failure), and all others were below 0.035 (Table 1).

### All-cause dementia

**Figure 1:**
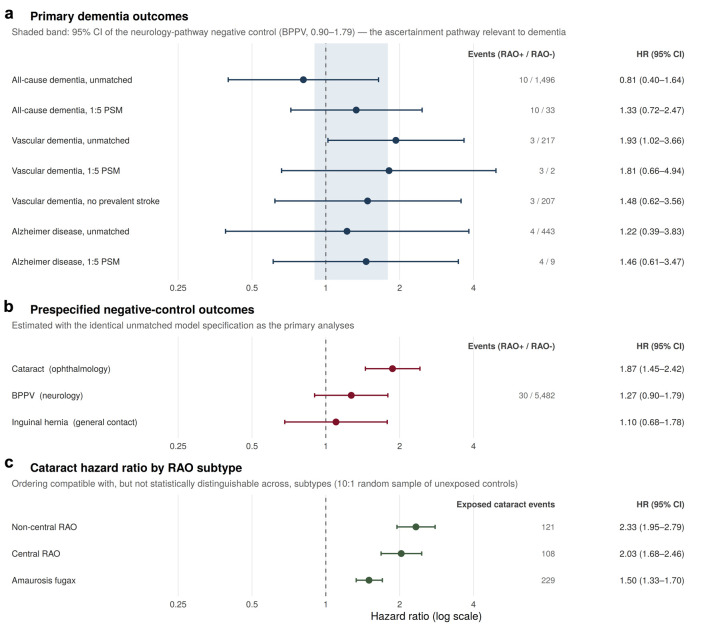
Hazard ratios (HRs) for primary and negative-control outcomes. Forest plot of HRs with 95% confidence intervals, log scale, with a vertical reference line at HR = 1. (a) Primary dementia outcomes: all-cause dementia unmatched 0.81 and matched 1.33; vascular dementia unmatched 1.93, matched 1.81, and with prevalent stroke excluded 1.48; Alzheimer disease unmatched 1.22 and matched 1.46. (b) Prespecified negative-control outcomes: cataract 1.87 (ophthalmology pathway), benign paroxysmal positional vertigo (BPPV) 1.27 (neurology pathway), inguinal hernia 1.10 (general healthcare contact). (c) Cataract HRs by retinal artery occlusion (RAO) subtype: non-central RAO 2.33, central RAO 2.03, amaurosis fugax 1.50. The shaded band in panel (b) marks the confidence interval of the neurology-pathway control (0.90–1.79), which is the pathway relevant to dementia ascertainment and therefore the appropriate calibrator for the primary outcomes in panel (a).

RAO was not associated with all-cause Wilkinson-confirmed dementia in unmatched (HR 0.81, 95% CI 0.40–1.64; P = 0.56) or 1:5 propensity-matched (HR 1.33, 95% CI 0.72–2.47; P = 0.37) analyses, on 10 exposed events (Fig. 1, Table 2). Under the broad RAO definition, the estimate was similar (HR 0.88, 95% CI 0.53–1.45; P = 0.62), as was the CRAO-only subgroup (HR 0.70, 95% CI 0.23–2.18; P = 0.54). The proportional hazards assumption was supported (treatment × log-time interaction P = 0.797). The matched estimate exceeded the unmatched estimate because matching removed imbalance in calendar year of cohort entry and sex, the two covariates with pre-match SMDs above 0.30; matching on these variables shortened the effective exposure-time advantage of the RAO+ group. Both intervals are wide and are compatible with a range of effects in either direction.

**Table 2:** Primary Outcomes, Sensitivity Analyses, and Prespecified Negative-Control Outcomes

Outcome and specification	Events, RAO+/RAO−	HR (95% CI)	P
All-cause dementia, unmatched	10/1,496	0.81 (0.40–1.64)	0.56
All-cause dementia, 1:5 PSM	10/33	1.33 (0.72–2.47)	0.37
Vascular dementia, unmatched	3/217	1.93 (1.02–3.66)	0.045
Vascular dementia, 1:5 PSM	3/2	1.81 (0.66–4.94)	0.25
Vascular dementia, no prevalent stroke, unmatched	3/207	1.48 (0.62–3.56)	0.38
Alzheimer disease, unmatched	4/443	1.22 (0.39–3.83)	0.73
Alzheimer disease, 1:5 PSM	4/9	1.46 (0.61–3.47)	0.40
Broad RAO, all-cause dementia, unmatched	-	0.88 (0.53–1.45)	0.62
CRAO only, all-cause dementia, unmatched	-	0.70 (0.23–2.18)	0.54
Negative control: cataract (ophthalmology), unmatched	-	1.87 (1.45–2.42)	< 0.001
Negative control: BPPV (neurology), unmatched	30/5,482	1.27 (0.90–1.79)	0.17
Negative control: inguinal hernia (general contact), unmatched	-	1.10 (0.68–1.78)	0.69
Cataract by subtype: non-central RAO, unmatched	121	2.33 (1.95–2.79)	< 0.001
Cataract by subtype: central RAO, unmatched	108	2.03 (1.68–2.46)	< 0.001
Cataract by subtype: amaurosis fugax, unmatched	229	1.50 (1.33–1.70)	< 0.001

All unmatched estimates derive from Cox proportional hazards models on an age timescale with left truncation at cohort entry and an identical covariate set (sex, calendar year of cohort entry, and baseline hypertension, diabetes, hyperlipidemia, atrial fibrillation, heart failure, stroke, transient ischemic attack, depression, and chronic kidney disease). Negative controls were not propensity-matched and are therefore compared with the unmatched primary estimates. Cataract subtype analyses used a 10:1 random sample of unexposed participants; the events column for those rows shows exposed events only. Bonferroni-corrected P for unmatched vascular dementia was 0.225 across the five prespecified primary tests (α = 0.01); the Benjamini–Hochberg adjusted value was also 0.225. BPPV: benign paroxysmal positional vertigo; CI: confidence interval; CRAO: central retinal artery occlusion; HR: hazard ratio; PSM: propensity-score matching; RAO: retinal artery occlusion.

### Dementia subtypes

Vascular dementia showed an unmatched HR of 1.93 (95% CI 1.02–3.66; P = 0.045) on three exposed and 217 unexposed events. The point estimate remained elevated after propensity matching (HR 1.81, 95% CI 0.66–4.94; P = 0.25; three exposed and two unexposed events) and after exclusion of participants with prevalent stroke (HR 1.48, 95% CI 0.62–3.56; P = 0.38; three exposed and 207 unexposed events). The widening of these intervals reflects the small number of exposed events rather than migration of the estimate toward the null. Under Bonferroni correction across the five prespecified primary tests, the unmatched vascular dementia P value was 0.225, as was the Benjamini–Hochberg adjusted value; we note that this is a statement about a decision threshold in an underpowered subgroup and not evidence that the effect is absent. Alzheimer disease was not associated with RAO in unmatched (HR 1.22, 95% CI 0.39–3.83; P = 0.73) or matched (HR 1.46, 95% CI 0.61–3.47; P = 0.40) analyses.

### Negative-control calibration

Cataract, the ophthalmology-pathway control, was substantially elevated (HR 1.87, 95% CI 1.45–2.42; P < 0.001), with an E-value of 2.26 for the lower confidence bound. Neither the neurology-pathway control BPPV (HR 1.27, 95% CI 0.90–1.79; P = 0.17; 30 exposed and 5,482 unexposed events) nor the general-contact control inguinal hernia (HR 1.10, 95% CI 0.68–1.78; P = 0.69) was elevated. Point estimates ordered by ascertainment-pathway intensity: ophthalmology 1.87, neurology 1.27, general contact 1.10. By RAO subtype, cataract HRs were non-central RAO 2.33 (95% CI 1.95–2.79), CRAO 2.03 (95% CI 1.68–2.46), and amaurosis fugax 1.50 (95% CI 1.33–1.70), all P < 0.001; this is an ordering compatible with, but not statistically distinguishable across, subtypes.

## Discussion

In a large, diverse, contemporary cohort within the All of Us Research Program, we found no evidence that RAO is independently associated with incident dementia after adjustment for shared vascular risk and time-varying confounding. This replicates, in a United States EHR population, the conclusion of the largest prior study of RAO as an isolated exposure, which reported a fully adjusted all-cause dementia HR of 0.98 in a Danish national cohort of more than two million individuals [2]. Convergence of a small, diverse, EHR-based United States cohort and a large, complete, register-based European cohort is more informative than either alone, because the two differ in nearly every source of bias except the biology.

The prespecified vascular dementia subtype requires a more careful reading. Its point estimate remained elevated across every specification we fitted, from 1.93 unmatched to 1.81 matched to 1.48 after prevalent-stroke exclusion, while the CIs widened as the analytic set shrank. With three exposed events, these data cannot distinguish a real vascular effect from chance, and a persistently elevated but imprecise estimate is at least as consistent with a true, underpowered vascular effect as with differential ascertainment. We therefore do not claim to have excluded a vascular dementia association. Notably, the Danish cohort found the same pattern with far greater precision: a crude vascular dementia HR of 1.61 that fell to 1.12 (95% CI 0.95–1.33) after comorbidity adjustment [2]. That study’s precision, rather than ours, is what makes a large independent vascular effect unlikely.

The methodological contribution of this study is the use of pathway-specific negative-control outcomes to quantify differential ascertainment within the dataset generating the primary estimate. The result is instructive. Detection bias in this cohort was large but pathway-specific: the ophthalmology control was elevated by 87%, whereas the neurology and general-contact controls were not elevated at all. Because dementia is ascertained through neurology, cognitive, and primary-care contact rather than through ophthalmology, the appropriate calibrator for a dementia outcome is BPPV (1.27) or hernia (1.10), not cataract. Calibrating against the same-pathway control implies that measurable detection bias accounts for only part of the residual vascular dementia estimate; the remainder is not explained by our negative controls. We consider this the honest reading of our own framework, and we note that the ordered subtype estimates do not straightforwardly support a contact-intensity account either: central RAO, an American Heart Association stroke equivalent, would be expected to trigger more rather than less ophthalmologic follow-up, yet its cataract HR was slightly lower than that of non-central RAO, with overlapping intervals.

The E-value of 2.26 for the lower bound of the cataract estimate indicates that an unmeasured confounder would need to be associated with both RAO and cataract by a risk ratio of at least 2.26, above and beyond the measured covariates, to explain that association away. We emphasize that the E-value addresses unmeasured confounding and not differential ascertainment specifically; it establishes that the cataract signal is not readily explained by a moderate unmeasured confounder, which leaves differential ascertainment as one plausible explanation among others rather than a demonstrated one.

The broader implication is that negative-control calibration should be routine in EHR studies linking retinal vascular events to neurologic, cognitive, or psychiatric outcomes, and that the control must be matched to the ascertainment pathway of the outcome under study. Reports of elevated dementia risk after retinal vein occlusion [11], an exposure that entails serial intravitreal therapy and repeated retinal imaging, are an immediate candidate for such calibration. A single generic negative control would have been misleading here in either direction: cataract alone would have overstated the detection bias relevant to dementia, and hernia alone would have understated the ascertainment intensity that RAO patients genuinely experience.

### Limitations

The exposed cohort is small (n = 339 strict) with few subtype events, and the study is underpowered to exclude clinically meaningful effects; the matched all-cause interval of 0.72 to 2.47 and the matched vascular dementia interval of 0.66 to 4.94 are compatible with the effect sizes reported in the prior literature. Competing mortality was not modelled: the analytic dataset did not include vital status, so we report cause-specific hazards throughout. The cause-specific hazard is the appropriate estimand for the etiologic question of whether RAO contributes to dementia, whereas a subdistribution hazard would answer a prognostic question about absolute risk in the presence of death; nonetheless, unmodelled competing mortality in an older, higher-comorbidity exposed group biases observed dementia incidence downward, and we cannot exclude this as a contributor to the all-cause null. Body mass index, smoking, alcohol use, educational attainment, race, ethnicity, and medication exposures were not available in the analytic dataset; several of these are established modifiable dementia risk factors [12]. Smoking is of particular concern as a shared cause of retinal arterial occlusive disease and vascular cognitive impairment; its omission would be expected to inflate rather than attenuate an RAO–dementia association, which limits interpretation of the residual vascular dementia estimate more than it threatens the null all-cause finding. All of Us enrollment is voluntary and may not generalize to the broader United States population. Outcome ascertainment relies on EHR coding, and dementia is both underdiagnosed and undercoded. Sex was derived from gender-identity concepts, which are not interchangeable with sex assigned at birth; any resulting misclassification is expected to be small and non-differential with respect to RAO status. Finally, the proportional hazards diagnostic and the cataract subtype analysis used random samples of unexposed participants for computational tractability, as stated in the “Methods”.

### Clinical implications

Patients with RAO carry substantial vascular burden and warrant aggressive secondary prevention on established cardiovascular grounds [1]. These data do not support routine post-RAO cognitive screening as a distinct intervention, but neither do they exclude a vascular dementia effect of the magnitude that would justify one; adequately powered cohorts are needed.

### Conclusions

We found no evidence that RAO is independently associated with incident dementia in the All of Us cohort, consistent with the largest prior study of RAO as an isolated exposure. The cohort was underpowered to exclude clinically meaningful effects, particularly for vascular dementia, where the point estimate remained elevated across all specifications. Detection bias in this dataset was demonstrably pathway-specific and large along the ophthalmology pathway, but calibration against the ascertainment pathway relevant to dementia accounts for only part of the residual vascular dementia estimate.

## Data Availability

The data supporting the findings of this study were obtained from the All of Us Research Program Researcher Workbench. These data are not publicly available due to participant privacy protections and data use agreements but may be accessed by qualified researchers through the All of Us Research Program following approval and completion of required training and agreements.
